# The Application of in vivo laser scanning confocal microscopy as a tool of conjunctival in vivo cytology in the diagnosis of dry eye ocular surface disease

**Published:** 2010-11-19

**Authors:** Takashi Kojima, Yukihiro Matsumoto, Murat Dogru, Kazuo Tsubota

**Affiliations:** 1 Johnson & Johnson Department of Ocular Surface and Visual Optics, Keio University School of Medicine, Tokyo, Japan; 2 Department of Ophthalmology, Keio University School of Medicine, Tokyo, Japan

## Abstract

**Purpose:**

To evaluate the applicability of in vivo laser scanning confocal microscopy as a tool of conjunctival cytology in a prospective case-control study.

**Methods:**

Nineteen right eyes of 19 Sjogren’s syndrome dry eye patients (19 females; mean age: 55.8±15 years), and 18 right eyes of 18 normal healthy control subjects (12 females and 6 males; mean age: 50.8±14 years) were evaluated in this study. The eyes were analyzed by the Heidelberg retina tomography (HRTII)/Rostock cornea module (RCM). Ocular surface and tear function tests including vital stainings (fluorescein and Rose Bengal), Schirmer test, tear film break up time (BUT), and conjunctival impression cytology were performed. After obtaining the confocal microscopy images, the mean individual epithelial cell area (MIECA), and nucleocytoplasmic (N/C) ratio were analyzed. The correlation between confocal microscopy and impression cytology parameters was also investigated.

**Results:**

The BUT, Schirmer test values, vital staining scores and squamous metaplasia grades in impression cytology were significantly worse in dry eye patients compared to controls (p<0.0001). The MIECA and the mean N/C ratios were worse in dry eye subjects compared to controls both in impression cytology and in vivo confocal microscopy (p<0.0001) with no significant differences between these parameters when the two examination techniques were compared. The MIECA and N/C ratio in conjunctival impression cytology showed significant correlation with the corresponding confocal microscopy parameters (MIECA, r^2^:0.557 ; N/C, r^2^:0.765).

**Conclusions:**

Laser scanning confocal microscopy seems to be an efficient non-invasive tool in the evaluation of phenotypic alterations of the conjunctival epithelium in dry eye disease. N/C ratio and MIECA appear to be two promising and new parameters of in vivo confocal cytology in the assessment of the ocular surface in dry eye disease.

## Introduction

Dry eye disease is a multifactorial disease of the tears and ocular surface that results in symptoms of discomfort, visual disturbance, and tear film instability with potential damage to the ocular surface [1]. Based on data from the largest epidemiologic studies of dry eye to date, the Women’s Health Study [2],  and other studies [3,4],  it has been estimated that about 3.23 million American women 50 years and older have dry eye disease [5]. A recent study estimated the prevalence of definite dry eye disease to be 10.1% in male subjects and 21.5% in female subjects in Japan [6].

Among entities causing dry eye disease, Sjogren`s syndrome (SS) is a multifactorial autoimmune disorder, mainly affecting the salivary and lacrimal glands, which is influenced by genetic as well as environmental factors that are not yet completely understood. SS occurs worldwide and in people of all ages. The peak incidence has been reported to be in the fourth and fifth decades of life, with a female-to-male ratio of 9:1 [7]. The incidence of primary SS reported in the literature varies from less than 1:1,000 to more than 1:100 [8].

Dry eye is one of the pivotal events in SS [9], the diagnosis of which requires an algorithm of multiple tests including Schirmer test, tear film break up time (BUT), and ocular surface evaluation with fluorescein, Rose Bengal, or Lisamine Green staining. The ocular surface dryness in SS has been linked to lacrimal hyposecretion and the accompanying inflammatory reactions of the ocular surface are believed to result in gradual ocular surface epithelial damage [9]. The study of the conjunctiva at the cellular level by relatively invasive methods such as impression or brush cytology [10] with incorporation of immunohistochemistry staining or flow cytometry [11] for inflammatory markers revealed elevated conjunctival inflammation in SS patients. Among them, impression cytology examines the ocular surface epithelium with application of cellulose acetate filter material to the ocular surface to remove the superficial layers of the epithelium. The technique is easy to perform, and can be employed to observe the ocular surface epithelial cell changes over time. Impression cytology has been used for many ocular surface diseases including dry eye, atopic keratoconjunctivitis, ocular pemphigoid, and superior limbic keratoconjunctivitis (SLK) [12-15]. Two studies using impression cytology samples by Nelson [16] and Tseng et al. [17] reported a grading system for squamous metaplasia based on cell size, N/C ratio, and goblet cell densities. Squamous metaplasia of the conjunctival epithelium has been reported as an integral part of many ocular surface diseases associated with dry eyes [18]. The change in the extent of squamous metaplasia has been reported to be useful in the evaluation of treatment responses in dry eye syndromes [19-22].

Although impression cytology is effective, safe and almost a non-invasive technique, it has limitations. It can evaluate only the superficial layer of the conjunctival epithelium. It is hard to evaluate the extent of inflammatory cell infiltration under or within the epithelium. It is also possible that there are cells that are not picked up by the cellulose acetate filter.

Confocal microscopy is a new emerging non-invasive technology to evaluate the tissue structure and cell phenotype in vivo, which is useful as a supplementary diagnostic tool for the assessment of the histopathological processes in many ocular surface diseases and anterior-segment disorders including the in vivo examination of the cornea, bulbar and palpebral conjunctiva, and the meibomian glands [23-27].

In this study, we used confocal microscopy and impression cytology to evaluate the squamous metaplasia based on diagnostic parameters including the mean individual epithelial cell area (MIECA) and nucleocytoplasmic (N/C) ratio.

We evaluated and characterized the conjunctival findings in patients with SS using laser scanning confocal microscopy, and then compare the confocal microscopy parameters with the impression cytology findings. We also evaluated whether confocal microscopy examination could be an alternative for impression cytology in the assessment of squamous metaplasia in SS.

## Methods

### Subjects and examinations

Nineteen right eyes from 19 SS patients (19 females; mean age: 55.8±15.0 years), and 18 right eyes of 18 controls (12 females, 6 males; mean age 50.8±14 years) were evaluated in this prospective study. The diagnosis of the aqueous tear deficiency was made by the following criteria: 1) presence of symptoms of dry eye, 2) abnormality of the tear production as determined by the Schirmer test (<5 mm after 5 min), 3) presence of tear film instability (<5 s), and 4) positive ocular surface Rose Bengal and fluorescein vital staining. The SS diagnosis was made according to the Japanese consensus criteria. Informed consent was obtained from all subjects. The study was ethic board reviewed and conducted in accordance with the tenets of the Declaration of Helsinki. None of the patients had a history of Stevens-Johnson syndrome, chemical, thermal, or radiation injury; or any other systemic/ocular disorder or underwent any ocular surgery or had contact lens use that would create an ocular surface problem. All patients were using nonpreserved artificial tears for 8 weeks at the time of initial examination and underwent tear function and ocular surface examinations including confocal microscopy before institution of additional dry eye treatment. The results were compared with the same examination parameters performed on the healthy control subjects.

### Tear function tests and ocular surface vital staining

The standard tear film break-up time measurement was performed. 1% fluorescein dye was instilled into the conjunctival sac as previously reported [28,29]. The interval between the last complete blink and the appearance of the first corneal black spot in the stained tear film was measured three times and the mean value of the measurements was calculated. This was followed by staining with 1% Rose-Bengal solution. Fluorescein and Rose Bengal stainings of the ocular surface were noted and scored. Both fluorescein and Rose Bengal staining scores ranged between 0 and 9 points. Any score above 3 points was regarded as abnormal. For further evaluation of tears, the Schirmer test without anesthesia was performed. The standardized strips of filter paper (Alcon Inc., Fort Worth, TX) were placed in the lateral canthus away from the cornea and left in place for five minutes with the eyes closed [28]. Readings were recorded in millimeters of wetting for five min. A reading of less than 5 mm was referred as an aqueous deficiency.

### Impression cytology

The impression cytology specimens were obtained after administration of topical anesthesia with 0.4% oxybuprocaine. Strips of cellulose acetate filter paper (HAWP 01300; Millipore, Bedford, MA) that were soaked in distilled water for a few hours and dried at room temperature were applied on the nasal bulbar conjunctiva adjacent to the corneal limbus, pressed gently by a forceps, and then removed. The specimens were then fixed with 10% formaldehyde, stained with periodic acid–Schiff, dehydrated in ascending grades of ethanol and then with xylene, and finally coverslipped. The quantitative studies of conjunctival epithelial cells were conducted by taking photographs with a calibrated grid under a light microscope at a magnification of 200×. We photographed ten different areas of each sample selected at random. We calculated the MIECA and N/C ratio using Image J software (National Institute of Health, Bethesda, MD) and averaged the outcomes.

### In vivo laser scanning confocal microscopy

In vivo laser confocal microscopy was performed on all subjects with a new generation confocal microscope, the Rostock Corneal Software Version 1.2 of the HRTII-RCM (Heidelberg Retina Tomograph II- Rostock Cornea Module; Heidelberg Engineering GmbH, Dossenheim, Germany). After topical anesthesia with 0.4% oxybuprocaine, the subject’s chin was placed on the chin rest. The objective of the microscope was an immersion lens covered by a polymethylmethacrylate cap (Tomo-Cap; Heidelberg Engineering GmbH). Comfort gel (Bausch&Lomb GmbH, Berlin, Germany) was used as a coupling agent between applanating lens cap and the ocular surface. After an examiner asked the patient to look straight into a pointer light source, the center of the Tomo-Cap was applanated onto the nasal bulbar conjunctiva by adjusting the controller, and the digital images of the underlying conjunctiva could be observed on the computer screen. When the first superficial conjunctival cells were visualized, the digital micrometer gauge was set at zero, and then by pressing on the foot pedal, sequence images were recorded by a charge-coupled device (CCD) color camera (maximum 30 frames/s) while gradually moving the focal plane into the subconjunctival tissue. The nasal conjunctiva was scanned while moving the applanating lens from the limbal area toward the caruncle with minute horizontal movements. Ten sequences each containing 100 frames were taken in each eye. Ten non-overlapping frames with the best resolution were selected from each sequence. We developed two new parameters, the mean individual epithelial cell area (MIECA) and N/C ratio, to evaluate the morphological changes of the conjunctival epithelium in SS in this study. The calculation of all these parameters were performed using Image J software. The length of a single confocal microscopy examination session was approximately 10 min. None of the subjects complained of discomfort nor any adverse effect was observed after an examination in this series.

### Statistical analyses

The Pearson’s correlation analysis was performed to analyze the correlation between impression cytology and confocal microscopy parameters. Age and sex differences were studied by the χ^2^ analysis. The differences in tear function tests between SS patients and controls were tested by the Mann–Whitney test. A p value of less than 0.05 was considered statistically significant. Instat software (Graphpad software Inc., La Jolla, CA) for Macintosh was used for these analyses.

## Results

The results of the tear function tests, vital stainings and squamous metaplasia grades in impression cytology in patients and control subjects are shown in Table 1. In patients with SS, the mean Schirmer scores, tear film break up times, the mean fluorescein, and Rose Bengal scores and the mean conjunctival squamous metaplasia grades were significantly worse than the control subjects (p<0.0001).

**Table 1:** Comparisons of tear function tests, vital staining scores and squamous metaplasia grades between SS patients and control subjects.

**Examination**	**SS patient**	**Healthy control**
BUT (s)	3.1±1.1*	12.0±2.8
Schirmer test-1 (mm)	2.8±1.9*	17.5±1.4
Fluorescein score (pts)	4.7±2.6*	0.5±0.9
Rose Bengal score (pts)	4.3±1.7*	0.2±0.4
IC squamous metaplasia grade (Nelson’s)	1.9±0.9*	0.3±0.4
BUT, tear film break up time; SS, Sjogren syndrome; IC, impression cytology		

*p< 0.0001

**Figure 1:**
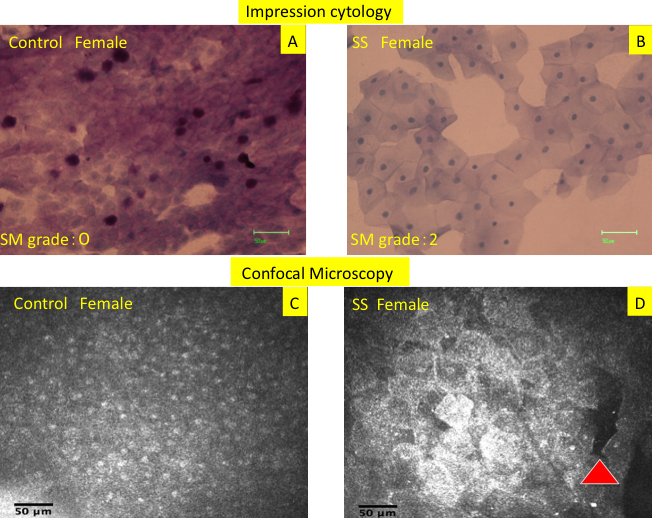
Impression cytology imprints and in vivo confocal microscopy scans from representative Sjogren syndrome patients and control subjects. **A**: Conjunctival impression cytology imprint showing sheets of epithelial cells with scanty cytoplasm and large nuclei in a 42 year old female control subject. Nelson’s squamous metaplasia grade: 0 Mean MIECA: 308 μm^2^ Mean N/C: 0.52. **B**: Imprint from a 38 year old female SS patient shows consistently large epithelial cells with abundant cytoplasm and pyknotic nuclei. Nelson’s squamous metaplasia grade: 2. Mean MIECA: 1799 μm^2^ Mean N/C: 0.18. **C**: Confocal microscopy scan showing sheets of densely packed small epithelial cells with large nuclei and scanty cytoplasm in the same control subject. Mean MIECA: 266 μm^2^ Mean N/C: 0.55. **D**: Confocal microscopy scan showing enlargement of individual superficial epithelial cells with pyknotic nuclei. Red arrow shows an area of cellular drop out. Mean MIECA: 1490 μm^2^ Mean N/C: 0.22.

Conjunctival impression cytology revealed sheets of epithelial cells with scanty cytoplasm and large nuclei in all control subjects whereas the imprints from SS patients showed consistently large epithelial cells with abundant cytoplasm and pyknotic nuclei. Imprints from representative subjects are shown in Figure 1A,B.

**Figure 2:**
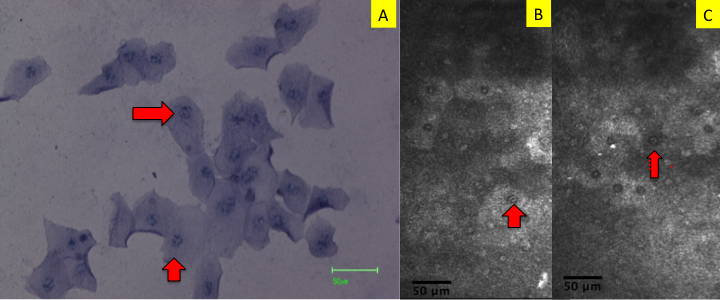
Impression cytology imprint and in vivo confocal microscopy scans from a representative Sjogren syndrome patient showing nuclear changes. **A**: Impression cytology imprint showing nuclear fragmentation and clumping(red arrows). **B**, **C**: Confocal microscopy scans showing these nuclear changes (red arrows).

Similarly, conjunctival confocal microscopy scans in normal subjects showed sheets of densely packed small epithelial cells with large nuclei and scanty cytoplasm whereas scans in patients with SS revealed that individual superficial epithelial cells were enlarged and had pyknotic nuclei. Areas of superficial epithelial cell loss were also observed in all patients. The representative confocal microscopy images are shown in Figure 1C,D. Nuclear fragmentation and clumping were also observed in corresponding impression cytology imprints and in vivo confocal microscopy scans of patients with SS (Figure 2 A-C).

### Correlation between confocal and impression cytology observations in relation to N/C ratio assessment

**Figure 3:**
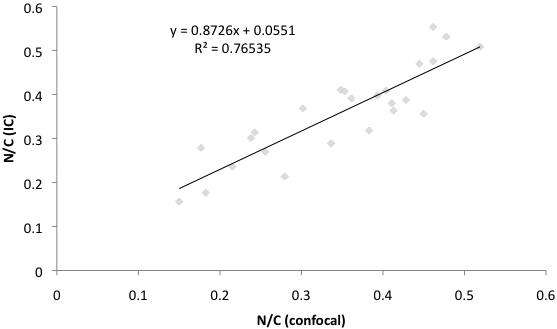
The correlation of nucleocytoplasmic (N/C) ratio assessed by impression cytology and in vivo confocal microscopy. Note the linear positive correlation for N/C ratio assessed by both examination techniques.

The N/C ratios with the confocal microscopy and impression cytology observation in the SS group were 0.34±0.11 and 0.34±0.01, respectively. Similarly, the N/C ratios in the control group were 0.47±0.06 and 0.45±0.05 with the confocal microscopy and impression cytology observations, respectively (Table 2). A significant correlation in relation to the N/C ratios was found between confocal microscopy and impression cytology methods in all subjects (r^2^=0.76535, p<0.0001; Figure 3).

**Table 2:** Comparisons of N/C ratios and MIECA values assessed by impression cytology and confocal microscopy between SS patients and control subjects.

	**Impression cytology**		**Confocal microscopy**	
**Squamous metaplasia parameter**	**Dry eye**	**Control**	**Dry eye**	**Control**
MIECA (μm)	945±574*	329±102	880±508*	378±119
N/C ratio	0.34±0.10*	0.45±0.05	0.34±0.11*	0.47±0.06
MIECA, mean individual epithelial cell area; N/C, nucleocytoplasmic				

*p< 0.0001

### Correlation between confocal microscopy and impression cytology observations in relation to MIECA assessment

**Figure 4:**
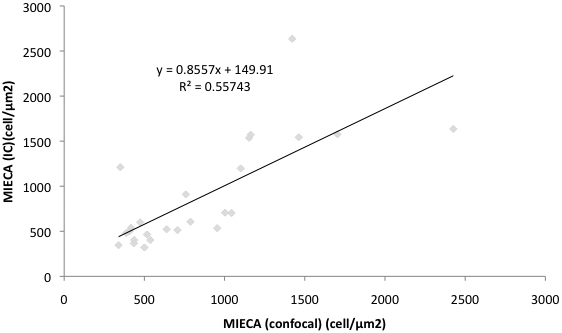
The correlation of mean individual epithelial cell area(MIECA) assessed by impression cytology and in vivo confocal microscopy. Note the linear positive correlation for MIECA assessed by both examination techniques.

The MIECA evaluated by the confocal microscopy and impression cytology observations in the SS group was 880±508 µm^2^ and 945±574 µm^2^, respectively. Similarly the MIECA evaluated by the confocal microscopy and impression cytology observation in the control group was 378±119 µm^2^ and 329±102 µm^2^, respectively. A significant correlation in relation to MIECA value was found between confocal microscopy and impression cytology methods in all subjects as shown in Figure 4 (r^2^=0.557, p<0.0001).

## Discussion

Squamous metaplasia has been reported to be associated with and be an important indicator of ocular surface health in various dry eye syndromes including Stevens Johnson syndrome, ocular pemphigoid, graft versus host disease, vitamin A deficiency, atopic keratoconjunctivitis, alkali injury related ocular surface disease, and Sjogren syndrome [17,18,30]. Decrease in N/C ratio, increased cell area, decreased expression of ocular surface mucins by both goblet and non-goblet cells, snake like chromatins, and pyknosis have been reported to be associated with squamous metaplasia [31]. The squamous metaplasia grades have been reported to be closely related to the severity of dry eye disease [17,32]. Nelson [16] and Tseng et al. [17] proposed grading systems using impression cytology samples which have been widely used in clinical practices.

While both impression cytology and tissue biopsies have been traditionally employed to study the process of squamous metaplasia and keratinization in conjunctival tissues and are very useful in the assessment of squamous metaplasia related changes, both are invasive techniques requiring removing of cells or tissue specimens.

Confocal microscopy has been recently reported by us to be an efficient tool in the assessment corneal and conjunctival changes in patients with Sjogren syndrome (SS) [33]. An important observation in that study was the significant decrease in the density of the superficial, intermediate and deeper conjunctival epithelial cells, which we thought might have been due to the elevation of the ocular surface inflammatory status and disturbances in the overall turnover of epithelial cells. Indeed, we could disclose presence of significant inflammation in the conjunctiva of patients with SS where inflammatory infiltrates consisted mainly of polymorphs. In addition, the study noted a significant increase in the density of epithelial microcysts in the conjunctiva of SS patients which was considered as another potentially useful clinical confocal microscopy parameter in the assessment of conjunctival disease and the responses to treatment [33].

In this study, we devised new confocal microscopy parameters namely, MIECA and N/C ratio, which we hoped would reflect the squamous metaplasia changes that have been traditionally described by impression cytology until now and thus compared these two parameters with the same parameters calculated from the impression cytology specimens. IC studies of the conjunctival epithelium in dry eyes showed keratinization as well as acanthosis, dyskeratosis, and changes of the nuclei including condensation of nuclear chromatin whose appearance is highly characteristic (snake like chromatin) together with cytoplasmic glycogen overload, pyknosis or nuclear loss during the process of squamous metaplasia [18]. Similarly, we noted higher squamous metaplasia grades, cellular enlargement, epithelial cells with pyknosis and nuclear changes including clumping and/or fragmentation, decreased intercellular cohesion in IC specimens of SS patients compared to controls. The mean MIECA and N/C ratios were found to be significantly higher in SS patients than controls with both IC and in vivo confocal microscopy examinations. The N/C ratios and MIECA values assessed by both methods showed a significant linear positive correlation in the subjects of the current study suggesting the possibility of application of in vivo confocal microscopy to the evaluation of the assessment of ocular surface health status in addition to impression cytology. Although prospective comparative studies involving larger number of dry eye patients are essential, the initial findings from this study also strengthen hopes that in vivo cytology by confocal microscopy may very well be applied to the evaluation of ocular surface treatment responses in dry eye clinical trials.

It should be noted that a decrease of goblet cells has been reported to be an integral part of the process of squamous metaplasia where this study refrained from describing the goblet cell alterations [17]. This is because of the current disagreement in relation to the appearance of goblet cells in the previously published studies which assessed the goblet cell densities by in vivo confocal microscopy [34,35]. While some in vivo confocal microscopy studies reported goblet cells as oval white bodies [35], others reported them as dark round structures [34]. Whether confocal microscopy images of brightly or darkly appearing goblet cells reflect pre or post secretion stages are still unclear and need further investigation.

In summary, the results of this study suggest that confocal microscopy may serve as a tool of in vivo cytology where N/C ratio and individual cell area appear to be two new promising parameters in describing the ocular surface health status through squamous metaplasia in patients with SS.
